# Bracing Patients with Idiopathic Scoliosis: Design of the Dutch Randomized Controlled Treatment Trial

**DOI:** 10.1186/1471-2474-9-57

**Published:** 2008-04-22

**Authors:** Eveline M Bunge, Harry J de Koning, the brace trial group

**Affiliations:** 1 Erasmus MC, University Medical Center Rotterdam, Dept. of Public Health, P.O. Box 2040, 3000 CA Rotterdam, The Netherlands

## Abstract

**Background:**

The effectiveness of bracing patients with IS has not yet been convincingly established due to a lack of RCTs. Some authors suggest that their results confirm that bracing is effective; others conclude that the effectiveness of bracing is doubtful or recommend a RCT. The aim of this study was to establish whether bracing patients with idiopathic scoliosis (IS) in an early stage will result in at least 5 degrees less mean progression of the curvature compared to the control group after two years of follow-up.

**Methods:**

A randomized controlled trial was designed. Eligible patients are girls and boys in the age group 8–15 years whose diagnosis of IS has been established by an orthopedic surgeon, who have not yet been treated by bracing or surgery, and for whom further growth of physical height is still expected based on medical examination and maturation characteristics (Risser ≤ 2). The Cobb angle of the eligible patient should either be minimally 22 and maximally 29 degrees with established progression of more than 5 degrees, or should be minimally 30 and maximally 35 degrees; established progression for the latter is not necessary. A total of 100 patients will be included in this trial. The intervention group will be treated with full-time Boston brace wear; the control group will not be braced. Every four months, each patient will have a physical and an X-ray examination. The main outcomes will be the Cobb angle two years after inclusion and health-related quality of life.

**Discussion:**

The results of this trial will be of great importance for the discussion on early treatment for scoliosis. Furthermore, the result will also be important for screening for scoliosis policies.

**Trial registration:**

Nederlands Trialregister ISRCTN36964733

## Background

Idiopathic scoliosis (IS) is defined as a lateral curvature of the spine of unknown origin with a minimal Cobb angle of 10 degrees. The Cobb angle is the angle between the upper most inclined vertebra and the lower most inclined vertebra. Besides a lateral curvature of the spine, there is a fixed rotation of one or more vertebrae and a rotational deformation of that vertebra. Once the curvature shows progression and exceeds a Cobb angle of about 20 to 25 degrees, the curvature is not likely to disappear spontaneously. Progression of the curvature usually occurs just before or during the growth spurt [1].

IS patients who are not fully grown and who have Cobb angles over 20–25 degrees with established progression of at least 5 degrees are usually treated with a brace [1]. A brace is a close fitting device applied to the trunk to try and prevent further progression of the curvature and thereby ultimately the need for surgical treatment. Surgical treatment may become necessary when not fully grown patients reach Cobb angles over 45–50 degrees [1].

Although many orthopedic surgeons feel that bracing might slow down progression of IS and some believe that reduction of the lateral curvature is also feasible, the effectiveness of early treatment of IS with a brace has not yet been established. No randomized controlled trial (RCT) on bracing for IS can be found in the Cochrane library or in PubMed. The majority of the studies are retrospective cohort studies. Some authors suggest that their results confirm that the brace is effective [2-4]; others conclude that the effectiveness of bracing is doubtful or recommend a RCT [5-8]. Some studies were performed without a control group; these study results are compared with results on the natural history from other studies. Another problem in the interpretation of the results of these studies is that they lack consistency regarding both inclusion criteria and the definitions of brace effectiveness [9]. In practice, there is currently no trial evidence that bracing IS patients is better than observation and watchful waiting [10-12]. Several Dutch orthopedic surgeons have reached consensus that a RCT is justified under the condition that the patients included are in an early stage of the clinical course of scoliosis [5]. In collaboration with these orthopedic surgeons, we formulated the current study design.

At first, serious doubts about this RCT were expressed, particularly regarding the willingness of IS patients to go through the process of randomization. In a preliminary study on the feasibility of a RCT on brace treatment for IS, we investigated the willingness of IS patients to accept the process of randomization. We tested the hypothesis that over 50% of IS patients with an early stage of IS visiting an orthopedic outpatient clinic, but not yet treated with a brace for this disorder, would be willing to participate in a RCT. The opinion of their parents was also explored. Parents of 30 patients were invited to participate in this pilot study, of which 21 (70%) agreed to do so. Patients and their parents were interviewed after receiving written information about the principle of randomization and the advantages and disadvantages of participating in a RCT on the effectiveness of brace treatment. This information was also verbally clarified at the beginning of the interview. In total, 87% of the patients (95%CI: 57% – 91%) and 70% of the parents (95%CI: 48% – 85%) agreed to participate in a RCT on bracing [13]. Table 1 shows the odds ratio for willingness to participate for some patient and parent characteristics. Fathers with a higher secondary education were borderline significantly more willing to participate in a RCT.

**Table 1:** Odds ratios (OR) and 95% Confidence Intervals (C.I.) for willingness of parents to let their child participate in a RCT for treatment with bracing

Variables		Willingness of parents to agree with participation		OR (95% C.I.)
		Yes	No	
Age	per 1 year rise in age, in the range 9 to 15 years			1.28 (0.76–2.16)
Gender	boy girl	6 8	2 5	1.88 (0.27–13.12)
Native country father	Netherlands Other countries	12 2	3 4	8.00 (0.96–66.44)
Native country mother	Netherlands Other countries	13 1	4 3	9.75 (0.78–121.96)
Educational level father	=> higher secondary education < higher secondary education	9 5	1 6	10.75 (0.99–116.6)
Educational level mother	=> higher secondary education < higher secondary education	7 7	1 6	5.99 (0.56–63.53)

## Methods

### Setting

Orthopedic surgeons of 10 Dutch hospitals (3 teaching and 7 non-teaching hospitals) are willing to participate in this trial. Orthopedic surgeons will verbally inform all new patients who meet the inclusion criteria about the trial and give them written information (see table 2). Patients and their parents are asked for an informed consent. Together these orthopedic surgeons are expected to recruit 100 patients in a one-year period. All participating orthopedic surgeons have extensive experience with brace treatment in patients with IS.

**Table 2:** Schematic overview of written information for (parents of) patients with idiopathic scoliosis

**Subject**	**Short explanation**
Background	- explanation what idiopathic scoliosis is - treatment options in early stages: brace or observation - effectiveness of brace treatment not established yet
Purpose	- establish whether early treatment with a brace prevents curve progression - evaluate the influence of treatment (brace vs. observation) on health-related quality of life
Design	- randomly assigned to brace or observation group by computer, no influence of patient, parent, orthopedic surgeon or researcher. 50–50% chance - all patients examined every four months, both groups same protocol - 4 times asked to fill in some questionnaires
Advantages and disadvantages	- not known yet who will have most advantages. brace group: maybe treated with effective brace; control group: delayed/no uncomfortable treatment of which effect has not been established
Risks	- both patients treated with a brace as patients being observed have risk on surgery; not yet known whether the risk is different between the groups - regular check-up; in case of progression > 10 degrees in patient in control group, the patient is available for brace
Closure	- end results after two years - depending on the results, again asked to give permission for follow-up until maturity
Voluntary participation	- participation is voluntarily, in case of refusal, the usual treatment in that hospital will be provided - can withdraw permission at any time of the study
Costs and incentive	- no extra costs for patients - no extra incentive for orthopedic surgeons for participation of a certain patient
Confidentiality	- all data will be treated confidentially - no names or data that can lead to identification will be used in rapports etc. - protocol was approved by medical ethical review board
Insurance	- Since no other treatment than currently used in the Netherlands is applied, an extra insurance for patients' safety was not necessary
Further information	- contact information of the researcher and an independent physician was supplied
Complaints	- contact information of a committee that handles complaints about the study

### Participants

Eligible patients are girls and boys in the age group 8–15 years whose diagnosis of IS has been established by an orthopedic surgeon, who have not yet been treated by bracing or surgery, and for whom further growth of physical height is still expected based on medical examination and maturation characteristics (Risser sign) established by X-ray. To expect further growth of physical height, only patients with a Risser sign ≤ 2 will be included. All participating orthopedic surgeons agreed that the Cobb angle of the eligible patient should either be minimally 22 and maximally 29 degrees with established progression of more than 5 degrees, or should be minimally 30 and maximally 35 degrees; established progression for the latter is not necessary. At inclusion of the study, data on calendar age, gender, height, maturation characteristics (Risser sign, menarche), size and location of the curvature will be recorded for all patients.

### Ethical Committee Approval

The Medical Ethical Review Board of the coordinating hospital approved this trial in December 2005 (MEC-2005-319). All other participating centers (n = 10) obtained approval from their local Medical Ethical Committee between March 2006 and June 2007.

### Intervention group

Early treatment of IS consists of wearing a brace that is intended to prevent the curvature from worsening. Patients in the intervention group will initially be braced for two years. The orthopedic surgeon will refer the patients to a qualified certified prosthetist orthotist who will measure, make and fit the brace. Each brace will be measured and modified individually for each patient to fit and correct her/his curvature. She/he will be advised to wear the brace every day for 18–23 hours. Boston braces will be used for all patients; this brace is used the most in the Netherlands. Patients are usually advised to attend physical therapy for muscle training and to correct body posture. Physical therapy alone is not expected to prevent further progression of the curvature [11]. Therefore, patients are free to choose whether or not they will attend physical therapy.

Although some orthopedic surgeons prefer to keep the patients in the hospital for a few days to allow them to become used to wearing the brace, others do not. The orthopedic surgeons are allowed to apply their own protocol concerning this hospital admission.

In case patients of the intervention group reach Cobb angles that require surgery, they can be operated.

### Control group

Patients in the control group will initially not be braced during the two study years, unless their curvature shows more than 10 degrees progression compared to the Cobb angle at inclusion. In this case, the orthopedic surgeon, patients and their parents could decide to start brace treatment. The patients in the control group are allowed to attend physical therapy if they want to, because physical therapy alone will not prevent further progression of the curvature.

In case patients of the control group reach Cobb angles that require surgery, they can be operated.

### Follow-up

Both the intervention and control group will follow the same protocol for monitoring the curvature during the two years of the study. Every four months the orthopedic surgeons will repeat the measurements of size and location of the abnormal curvature, physical height, maturation characteristics (menarche and Risser sign), brace compliance, and whether or not surgery is indicated. These examinations will be conducted following a specific protocol: every four months a physical examination and an X-ray of the spine will take place. Patients will take off the brace the night before an X-ray is taken. Standard technique is a standing position of the patient and a posteroanterior projection. In patients with discrepancy in leg length, boards will be put under the shortest leg to correct for this.

### Objectives

The purpose of this RCT is to establish whether brace treatment in IS patients with Cobb angles between 22 and 35 degrees significantly reduces further bending of the spine, thereby preventing surgery for some patients. The specific research questions are:

1. Will bracing patients with IS in an early stage result in at least 5 degrees less mean progression of the curvature compared to the control group after two years of follow-up?

2. Do differences in health-related quality of life exist between patients with IS who are treated with a brace and patients who are under watchful waiting?

3. If bracing proves to be effective, what is the cost-effectiveness of brace treatment, compared to regular surveillance only in terms of cost per avoided surgery and cost per QALY, accounting for the (increased) burden of bracing and the (reduced) burden of surgery? If bracing does not appear to be effective, what are the possible savings if treatment guidelines would be changed?

4. If bracing proves to be effective, what is the cost-effectiveness of the nationwide screening program for scoliosis?

### Outcomes

The primary outcome of this RCT is that bracing will be considered potentially effective, if after two years the mean progression of the abnormal curvature in the intervention group is at least 5 degrees less than in the control group. Two years after date of randomization the primary outcome measure, progression in Cobb angle, will be established in each patient. Mean Cobb angles, as reported independently, in the intervention arm will be compared to mean Cobb angles in the control arm. To reduce inter-observer measurement errors, two orthopedic surgeons associated with the project team will judge all X-rays independently and without knowledge of the allocation arm. The mean value of the observed Cobb angles will serve as assessed value, whenever the difference between the two measurements is less than 3 degrees. If the difference is larger, a third orthopedic surgeon will be asked to measure the Cobb angle; the mean Cobb angle of all 3 measurements will serve as the assessed value.

The secondary outcomes of this study concern health-related quality of life (HRQoL) and compliance to brace treatment. Because bracing is a burdensome procedure (including reduced activity in daily living, pain, and implications for self-esteem, satisfaction with appearance, and mood), HRQoL will be evaluated in the intervention and control group by a generic HRQoL questionnaire, i.e. Child Health Questionnaire-Child Form 87 items (CHQ-CF87) and the Child Health Questionnaire-Parent Form 50-items, (CHQ-PF50) EuroQol including a VAS for general health, and a disease-specific questionnaire, i.e. the adjusted Scoliosis Research Society-22 Patient Questionnaire for Idiopathic Scoliosis (SRS-22r Patient Questionnaire). These questionnaires have been translated into Dutch; score distribution and internal consistency corresponded with the original versions [14-16]. Patients will be asked to fill out the CHQ-CF87, EuroQol and SRS-22 Patient Questionnaire just before every other visit to the orthopedic surgeon, thus 4 times in total. The EuroQol scores can be translated into utilities with values from the Dutch general population. The parents of the patients are requested to fill out the CHQ-PF50, EuroQol and SRS-22 at the same time that their child fills out her/his questionnaires. Patients and parents will receive the questionnaires and a return envelope about two weeks before every other visit to the orthopedic surgeon; thus they can fill out the questionnaires before they know the results of the X-ray (i.e., before knowing whether or not the curvature has progressed).

IS patients are usually advised to wear the brace for 18–23 hours every day as long as the patient is growing, which often implies wearing the brace during many years in adolescence. Hence, the risk of non-compliance is present. Lack of compliance in this group would lead to an underestimation of the treatment effect, if present, and would reduce the power of the trial. Compliance will be measured by three different means. The orthopedic surgeon will ask the patients and their parents how many hours a day the patients wear the brace. Because the patient has to return every 4 months for a check-up, the brace will be checked for signs of wear and tear typical for an intensively-used brace. Besides this, patients of the intervention group will receive a short questionnaire in which compliance, attitudes, social influences and barriers to wearing the brace will be measured at every other visit to the orthopedic surgeon.

### Sample size

The Cobb angle at inclusion will on average be 29 degrees (range 22–35 degrees). Due to the standard error of radiographic production and intra and inter-observer measurement variation, a measurement error in Cobb angles of 5 degrees will appear [1]. To reduce the standard error of radiographic production, all patients will undergo X-ray following a strict protocol. Because inter-observer measurements errors are reduced (see *Outcomes*), measurement error will than be maximal 2 degrees, thus the 95% observation interval of Cobb angles at inclusion is 20–37 degrees. After two years, when the outcome will be determined, the range in change will be 0–15 ± 2 degrees. Assuming a uniform distribution of this change in Cobb angle between the patients after two years (most unfavorable scenario), the standard deviation of the difference in change between baseline and outcome measurement will be about 4.5 degrees.

Since bracing has to be better than observation to be justified as treatment for IS, a superiority design will be used. With a power of 95% and alpha = 0.05, a mean difference of 5 degrees between the groups can be detected with 40 patients in both study arms. We will aim at a study population at start of 100 patients to take loss to follow-up into account.

### Randomization

Patients and their parent(s) who decide to participate will be asked to return the informed consent form to the researchers. Upon receipt of this form, randomization will be performed centrally by the department of Public Health (Erasmus MC) using computer-generated lists. Lists will be constructed with randomly permuted blocks per stratum, where strata will be defined by the participating center. The orthopedic surgeon will notify the patients and their parents of the outcome of allocation to the intervention group or the control group.

### Blinding

In this study, blinding of patients and orthopedic surgeons for treatment is not possible. However, a proper, blindly and independently conducted judgment of the X-rays in both trial groups is essential for the primary outcome. Two orthopedic surgeons will judge all X-rays of the patients of both groups and calculate the Cobb angles. To ensure blinding of the primary outcome, the randomization status of the participants will not be disclosed to these two orthopedic surgeons, who judge the patient's X-rays.

### Statistical methods

The intervention and control group will be compared based on the 'intention to treat' principle. Differences in Cobb angle between the two groups and other continuous parameters will be measured using parametric or non-parametric tests (depending on skewness) for group comparisons. Categorical parameters will be compared by the Chi-square test. Logistic regression will be applied to measure the treatment effect (yes/no progression) adjusting for covariates. Since we have 4-month measurements on Cobb angles in both arms, progression in Cobb angle will be analyzed with a linear mixed effect model, where we will assume a simple compound symmetry structure. This may be particularly important for extrapolations to follow-up periods longer than 2 years after entry.

When a patient in the control group had to be braced before the end of the follow-up (because of a more than 10 degree progression of the curvature), her/his Cobb angle at the moment of commencing treatment will be considered as final outcome and will be included in the analysis.

In a second analysis, we will perform Kaplan-Meier analyses with these 'progression to more than 10 degrees' as events, in both arms (intention-to-treat).

Stratified analysis will be done to evaluate whether curve type, brace compliance, Cobb angle at inclusion and Risser sign at inclusion influence the effectiveness of brace treatment. Using logistic regression we will also evaluate whether brace compliance depends on gender and age.

Health-related quality of life and utilities will be compared between the intervention and control group. This will be done by basic descriptive statistics. Depending on the distribution of the data, parametric or non-parametric tests will be used.

## Discussion

In collaboration with Dutch orthopedic surgeons we have designed the first randomized controlled treatment trial and started it in 2006. In 2007, dr. Weinstein et al. also started a randomized controlled treatment trial on bracing in the USA (BrAIST) [17]. The results of these trials will be of great importance to the discussion on bracing patients with IS. At the moment, patients and parents face a dilemma, because the only available treatment has not been proven effective, and is a rather burdensome one.

The results of this study will also be valuable for the screening program for scoliosis. Screening aims at detecting scoliosis in an early stage of the clinical course to allow brace treatment to try and prevent further progression of the curvature and reducing the need for surgery [11]. Recently, we performed a case control study on the effectiveness of screening for scoliosis. In that study, the case group consisted of surgically treated IS patients (the condition screening and early treatment should prevent) and the control group consisted of a random sample of Dutch youth. We found no evidence that cases were significantly less screened than controls [18]. If we had found a positive effect of screening, that would have implied that bracing is effective. A RCT on the effectiveness of bracing now seems even more justified. If bracing shows to be effective, the screening program needs to be revised. If bracing doesn't prove to be effective, a screening program is not applicable, since the availability of an effective early treatment is one condition for a screening program to be justified [19].

## Competing interests

The author(s) declares that they have no competing interests.

## Authors' contributions

EB and HJdK designed the study protocol in cooperation with the brace trial group. EB drafted the manuscript, under supervision of HJdK. EB, HJdK and the brace trial group read and approved the manuscript.

## Pre-publication history

The pre-publication history for this paper can be accessed here:


